# Characterization of Silk Fibroin/Chitosan *3D* Porous Scaffold and *In Vitro* Cytology

**DOI:** 10.1371/journal.pone.0128658

**Published:** 2015-06-17

**Authors:** Shuguang Zeng, Lei Liu, Yong Shi, Junqi Qiu, Wei Fang, Mingdeng Rong, Zehong Guo, Wenfeng Gao

**Affiliations:** 1 Department of Oral and Maxillofacial Surgery, Guangdong Provincial Stomatological Hospital Affiliated to Southern Medical University, Guangzhou, 510280, Guangdong, P.R. China; 2 Department of Children stomatology, Branch Hospital of Stomatology, The Affiliated Zhongshan Hospital of Sun Yat-sen University, Zhongshan, 528403, Guangdong, P.R. China; Michigan Technological University, UNITED STATES

## Abstract

Bone tissue engineering is a powerful tool to treat bone defects caused by trauma, infection, tumors and other factors. Both silk fibroin (SF) and chitosan (CS) are non-toxic and have good biocompatibility, but are poor biological scaffolds when used alone. In this study, the microscopic structure and related properties of SF/CS composite scaffolds with different component ratios were examined. The scaffold material most suitable for osteoblast growth was determined, and these results offer an experimental basis for the future reconstruction of bone defects. First, via freeze-drying and chemical crosslinking methods, SF/CS composites with different component ratios were prepared and their structure was characterized. Changes in the internal structure of the SF and CS mixture were observed, confirming that the mutual modification between the two components was complete and stable. The internal structure of the composite material was porous and three-dimensional with a porosity above 90%. We next studied the pore size, swelling ratio, water absorption ratio, degradation and *in vitro* cell proliferation. For the 40% SF-60% CS group, the pore size of the scaffold was suitable for the growth of osteoblasts, and the rate of degradation was steady. This favors the early adhesion, growth and proliferation of MG-63 cells. In addition to good biocompatibility and satisfactory cell affinity, this material promotes the secretion of extracellular matrix materials by osteoblasts. Thus, 40% SF-60% CS is a good material for bone tissue engineering.

## Introduction

Scaffold materials are critical to bone tissue engineering. They not only deliver the seed cells and growth factors to the defect site, but also support new bone tissues. The use of two or more mutually modified materials to construct composite scaffolds has become an important trend. Specifically, silk fibroin (SF) and chitosan (CS) have unique advantages and have received much attention as scaffold materials that can promote osteogenic activity [1–5].

SF/CS is a popular material [6–8] because of its good tissue compatibility [9,10]. However, there have been relatively few studies on *in vitro* composite culture of osteoblasts MG-63 and SF/CS scaffold that examined the osteogenic properties of the SF/CS scaffold. Various studies involving SF/CS scaffolds have demonstrated that these scaffolds can be successfully characterized and used for growing cells, but these studies have involved cells other than those in the osteogenic lineages [11–14]. Furthermore, previous studies involving SF/CS scaffolds for bone tissue engineering have often prepared scaffolds through electrospinning [15–17]. However, electrospinning may produce limitations in porosity, thereby restricting cell growth [18], and increasing the thickness of the scaffold can be difficult. Finally, many previous studies examined only a single or very limited number of ratios of SF to CS [12,13,19,20]. Thus, the goal of this study was to prepare a stable and reliable 3D porous scaffold material suitable for osteoblasts through a freeze-drying method. The material must have good biocompatibility and cell affinity as well as the ability to sustain an osteoblast extracellular matrix and facilitate bone repair.

First SF and CS were mixed in different proportions and after several freeze-drying processes they were combined with chemical cross-linking. This gave porous scaffolds of defined strength. We examined their properties with electron microscopy (EM), porosity examination, infrared analysis, X-Ray diffraction analysis (XRD) analysis and energy-dispersive X-Ray spectroscopy (EDS) analysis. By comparing the water absorption ratio, swelling ratio and degradation ratio of the scaffolds, the ideal material suitable for growth of osteoblasts was determined. MG-63 cells were seeded on the SF/CS scaffold, and their growth, adhesion, and proliferation on the scaffold was examined with fluorescent staining. Finally, we studied the formation of mineralized nodules on the scaffold and the ability of the scaffold to promote the osteoblast cell line MG-63 to secrete alkaline phosphatase (ALP).

## Materials and Methods

### 1. Preparation of SF/CS scaffold and characterization of its structure and properties

#### (1) Preparation of SF/CS scaffold material

First, 5 g fibroin powder was dissolved in a three component calcium chloride dissolution system CaCl_2_:C2H_5_OH:H_2_O = 1:2:8 (molar ratio) [21] and subjected to magnetic stirring in a 80°C water bath until it was completely dissolved. After it was cooled to room temperature, the solution was poured into a dialysis bag with a molecular weight cut off (MWCO) of 7000–10000 Da and submerged in deionized water. The solution was dialyzed at 4°C for three days. The water was changed once every three hours to remove small molecules from the silk fibroin solution. The dialysis bag containing the SF solution was placed into polyethylene glycol 6000 powders, dried and concentrated to collect the liquid. This was centrifuged at 3,500 rpm for 15 min to remove the insolubles, and the supernatant was collected. Three samples were washed clean and dried at 80°C and then fully cooled and weighed (M_1_). In each weighing bottle, 5 ml SF solution was added and weighed again—this was denoted M_2_. The bottles were then placed in a 60°C oven for 12 h, and weighed after cooling (M_3_). The following formula was then used:

SF concentration % = (M_3_−M_1_)/(M_2_−M_1_)×100%

The mean of the three samples was calculated and determined to be 2–3%.

For preparation of CS solution, 3 g CS powder was dissolved in 100 ml 0.2 M acetic acid solution to give a 3% CS solution that was pale yellow with relatively high viscosity. This solution was poured into a Buchner funnel and vacuum filtered to remove impurities.

The prepared 2% SF solution and 3% CS solution was mixed in different ratios, so that the mass fractions of SF and CS were 0%, 100%; 20%, 80%; 40%, 60%; 60%, 40%; 80%, 20%; and 100%, 0%. The solutions in each of the six groups were poured into a Teflon mold, prefreezed at -20°C for 24 h before being placed into a -80°C freezer for 24 h, and finally into a freeze-drying machine for 48 h to prepare the preliminary SF/CS scaffold. The material was immersed in an anhydrous methanol and 10% sodium hydroxide (volume ratio 1: 1) solution and soaked for 24 h before lyophilization for 48 h. Then, the material was soaked in a 95% aqueous ethanol solution containing 50 mmol/l ethylene dichloride (EDC) and 18 mmol/l N-Hydroxysuccinimide (NHS). This was subjected to cross-linking at 4°C for 24 h. Excess crosslinking agent on the scaffold was washed and then freeze-drying was repeated for 48 h to obtain the final SF/CS scaffold material.

#### (2) The macroscopic appearance of the scaffold material was examined visually

Scaffold blocks were frozen with liquid nitrogen and then broken into pieces to expose the internal structure. After gold spraying, the internal structure and morphology of the cross sections were observed under EM.

#### (3) Measurement of porosity [22]

Scaffold materials (dry mass M_0_) were immersed in ethanol in a weighing bottle. The bottle was weighed before (M_a_) and after (M_b_) removing the wet scaffold. Meanwhile a 50 mL pycnometer filled with ethanol was weighed with the weight denoted as M_1_. The ethanol was poured out and the wet scaffold previously soaked in ethanol was placed in the pycnometer, and ethanol was added until the pycnometer was filled to the same mark. The pycnometer was weighed again with weight denoted as M_2_.

Porosity was calculated according to the following equations (ρ is the density of ethanol):

Volume of scaffold pores: V1 = (M _a_-M _b_-M _0_)/ρ,

Apparent volume of the scaffold: V2 = (M _a_-M _b_)- (M _2_-M _1_)/ρ,

Porosity of the scaffold: ε = [V_1_/V_2_)]×100% = (M_a_-M_b_-M_0_)/(M_a_- M_b_)-(M_2_- M_1_)×100%.

#### (4) Infrared anaylsis

Scaffold materials were shredded and mixed evenly with potassium bromide powder for infrared scanning.

#### (5) XRD analysis

The experimental instruments and parameters included a copper target, LynxExe array detector, 40 kV, 40 mA, scanning step of 0.04°, and a scanning speed of 35.4 s/step.

#### (6) EDS analysis

The EDS analyzer equipped with an environmental scanning EM was used for qualitative and quantitative element analysis of the cross-section of the sample material.

### 2. Experiments on the physical and chemical properties of the 3D porous SF/CS scaffold material and degradation test

#### (1) Water absorption rate

Scaffold was weighed and placed in deionized water for 24 h. The surface moisture was then removed with filter paper, and the weight M_1_ noted. Weight M_2_ was noted after drying. Water absorption rate was calculated according to the following equation:

Water absorption = (M_1_−M_2_)/M_2_×100%

#### (2) Swelling ratio

Scaffold material of volume V_1_ was placed in deionized water for 24 h, and the volume when hydrated was determined to be V_2_. The swelling ratio was calculated according to the following equation:

Swelling ratio = (V_2_-V_1_)/V_1_×100%

#### (3) Degradation property test

Sterilized simulated body fluid (SBF) was placed in a 50 ml plastic bottle that had been sterilized with ultraviolet rays. A scaffold block was weighed (denoted as W_0_) and placed in the SBF. The bottle was placed under a vacuum to remove air until the scaffold was completely immersed in the liquid, and then placed in a moisturizing thermostat at 37°C. We measured pH at 1 h, and at 1, 3, 6, 9, 12, 15, 22, 29, 36, 50, and 64 d. Samples were dried at 60°C and weighted (denoted as W_1_). The formula used was: Degradation Ratio = (W_0_-W_1_)/W_0_×100%.

### 3. Biocompatibility and osteogenic properties of the 3D SF/CS porous scaffold

#### (1) The groups of testing the biocompatibility and osteogenic properties

Based on the results of these initial experiments, the groups used for testing the biocompatibility and osteogenic properties of the 3D SF/CS porous scaffold were: experimental groups (100% CS group and 40% SF-60% CS group), and a blank control group (the slide group).

#### (2) Processing of the scaffold material

The scaffold in the 100% CS group and the 40% SF-60% CS group was prepared into a thin 1 cm square with a 1-mm thickness. The glass slide in the control group was also cut into a 1-cm square sheet. All materials were irradiated with Co^60^ for disinfection for 24 h. Before use, they were treated with ultraviolet rays for 30 min.

#### (3) Cell inoculation

The scaffold materials in the 100% CS group, the 40% SF-60% CS group and the control group were pre-wetted in culture medium for 24 h. Then, 100 μl of cell suspension (5×10^5^/ml cells except in the adhesion rate test, where 1×10^6^/ml cells were used) was dropped onto the scaffold material, and agitated for 5 min so that the cells were seeded uniformly in the pores of the 3D scaffold. The three groups were then placed in the same incubator for 1 h, and 200 μl medium was added along the edge of the well until all the scaffold materials were immersed. The scaffolds were kept in the incubator for continuous culture.

#### (4) Cell growth and morphplogy

We examined the growth and morphology of MG-63 cells on different scaffold materials at different time points with an inverted phase contrast microscope.

#### (5) Measurement of cell adhesion rate

100 μl cell suspension containing 1×10^5^ cells were seeded on the pre-wetted scaffold in a 24-well plate. At 1, 3 and 6 h after inoculation, the adhesion rates of the experimental groups and the control group were measured. For each group, samples were collected from three wells. The medium was aspirated and the cell number in the medium solution was calculated (A_1_). After removing the material, cells adhered to the well wall were digested and counted (A_2_). The adhesion rate was calculated using the following equation:

Adhesion rate = [(A_0_−A_1_−A_2_)/A_0_]×100% (where A_0_ is the number of seeded cells).

#### (6) Hoechst staining

A cell suspension (100 μl) of density 5×10^5^ was seeded on the pre-wetted scaffold in a 24-well plate. At 3, 6, and 12 h and 1, 2, and 3 d after inoculation, the scaffolds were removed, rinsed with PBS, and fixed with 4% paraformaldehyde for 10 min. After repeated rinses with PBS, 300 μl 10 μg/ml Hoechst solution was added, and incubated at room temperature for 30 min. After rinsing with PBS, the residual PBS on the surface was removed with filter paper, and the scaffold was immediately examined under a fluorescence microscope.

#### (7) Measurement of the cell proliferation rate

A cell suspension (100 μl) of density 5×10^5^ was seeded on the pre-wetted scaffold in a 24-well plate. At 1 d, 3 d, and 5 d after inoculation, the scaffolds were removed, rinsed, and transferred to a new 24-well plate, in which 300 μl fresh medium was added. Then 60 μl MTS was added in the dark, and the plate was placed in a 37°C incubator for 3 h. Two samples were collected from each well, 100 μl each time, and placed in a 96-well plate. The 96-well plate was then placed in a microplate reader to measure the absorbance (OD value) at 490 nm. The slide group was the blank control group.

#### (8) Measurement of ALP activity

A cell suspension (100 μl) of density 5×10^5^ was seeded on the pre-wetted scaffold in a 24-well plate for 7, 14, and 21d. Every other day, the plate was rinsed with PBS and 300 μl fresh medium was added. At each time point, the material was removed and rinsed with PBS before 300 μl Triton X-100 lysate was added. The material was placed in a 4°C refrigerator for 24h. Then two lysate samples were collected from each well, 100 μl each time, and placed in a 96-well plate. A solution of pNPP was added in the dark (200 μl per well), and the plate was placed in an incubator for 30 min. A microplate reader was used to measure the optical density (OD) value of each well at 450 nm. The slide group was the blank control group.

#### (9) Observation of the calcified nodules

A cell suspension (100 μl) of density 5×10^5^ was seeded on the pre-wetted scaffold in a 24-well plate. Every other day, the plate was rinsed with PBS and 300 μl fresh medium was added. Starting from day 7, the calcified nodules in the MG-63 cells were observed with light microscopy.

### 4. Statistical analysis

SPSS16.0 software was used for statistical analysis. The data are all expressed as means ± standard deviation. Factorial analysis of variance (ANOVA) was performed. For between-group comparison at each time point, one-way ANOVA was performed. For hypothesis testing, we used a two-sided test. The significance level *P* = 0.05, i.e., *P*<0.05 was considered statistically significant.

## Results

### 1. Preparation of SF/CS scaffold and characterization of its structure and properties

#### (1) Observation of the internal structure

**Fig 1:**
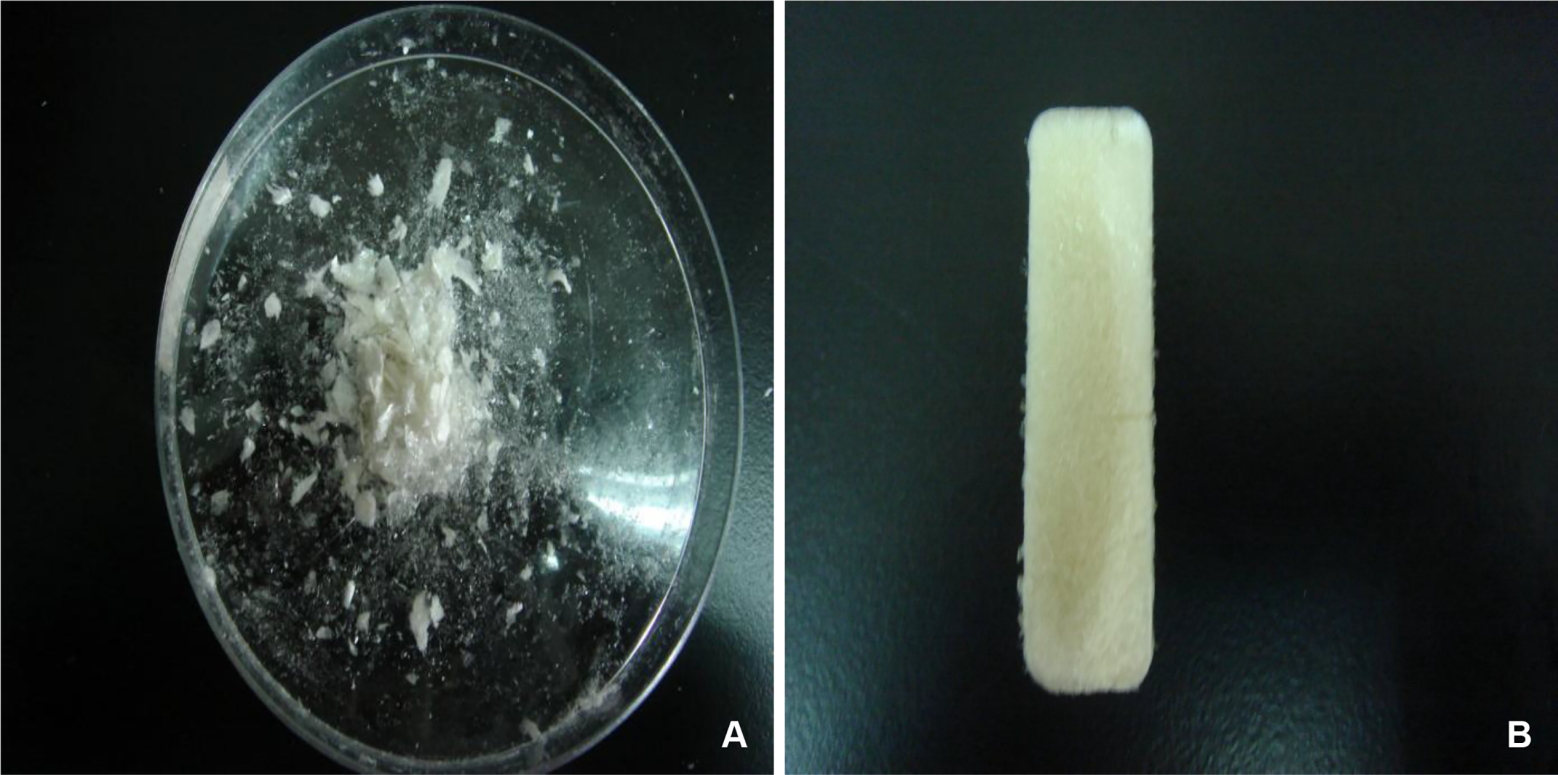
Pure SF and SF/CS scaffold after lyophilization. (A) pure SF in powder form is difficult to grip and has poor mechanical properties. (B) The SF/CS sample is yellowish white with a rough surface and porous internal structure.

Using the naked eye, we found the composite scaffold to be yellowish white with a rough surface and a loose, spongy, and flexible interior. Pure SF after lyophilization was in the powder form. It was difficult to handle and had poor mechanical properties (Fig 1).

**Fig 2:**
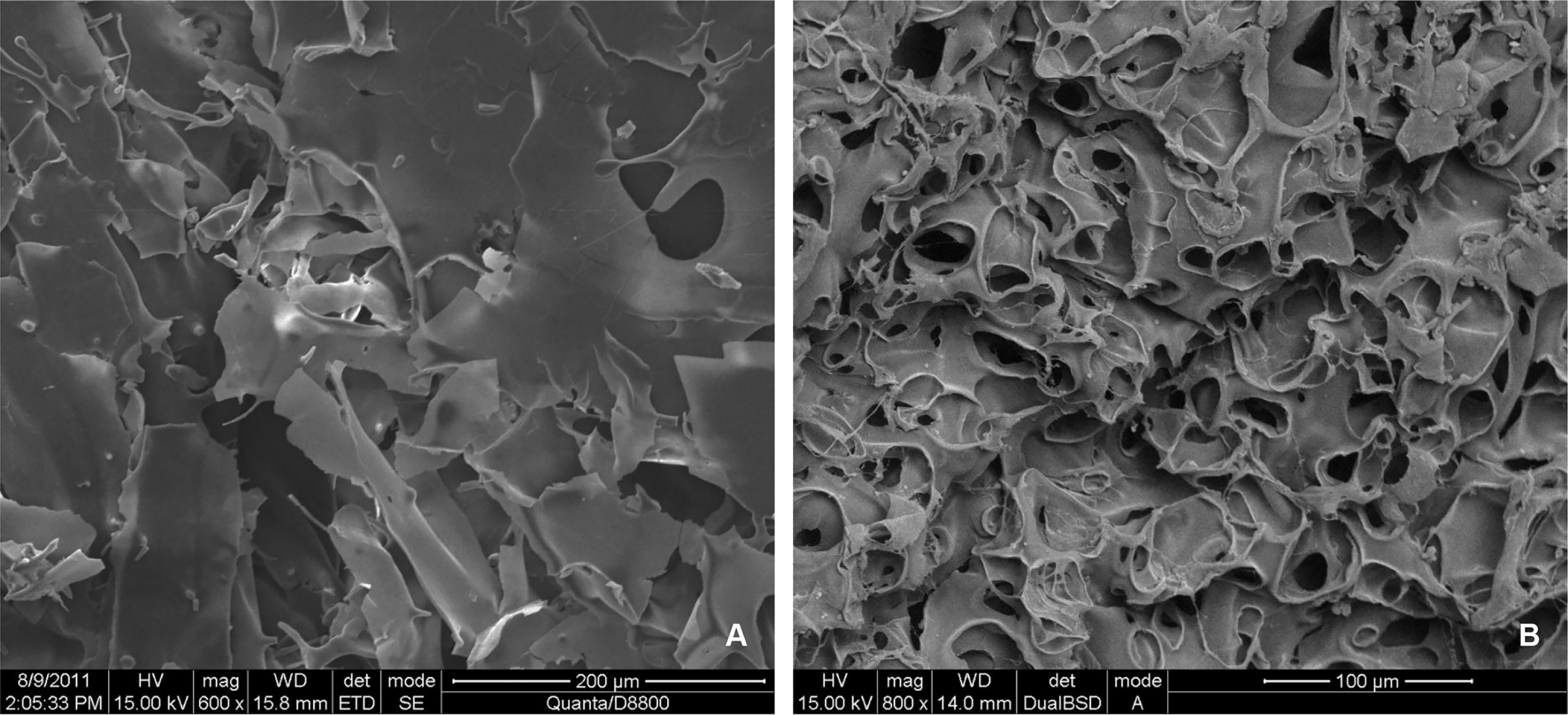
EM image of pure SF and pure CS. (A) pure SF after lyophilization showing a lamellar, curled structure with no typical pore structure. (B) pure CS after lyophilization with small pores, poor connectivity, and uneven sizes.

Under EM, pure SF (100% SF) scaffold was lamellar with a curled structure. There was no typical pore structure (Fig 2A). Pure CS (100% CS) had small pores with poor connectivity and non-uniform sizes (Fig 2B). Due to the poor mechanical properties of 100% SF scaffolds and lack of a porous structure, 100% SF was not used as a comparison in the water absorption and swelling ratio studies, nor was it used in studies involving MG-63 cells.

**Fig 3:**
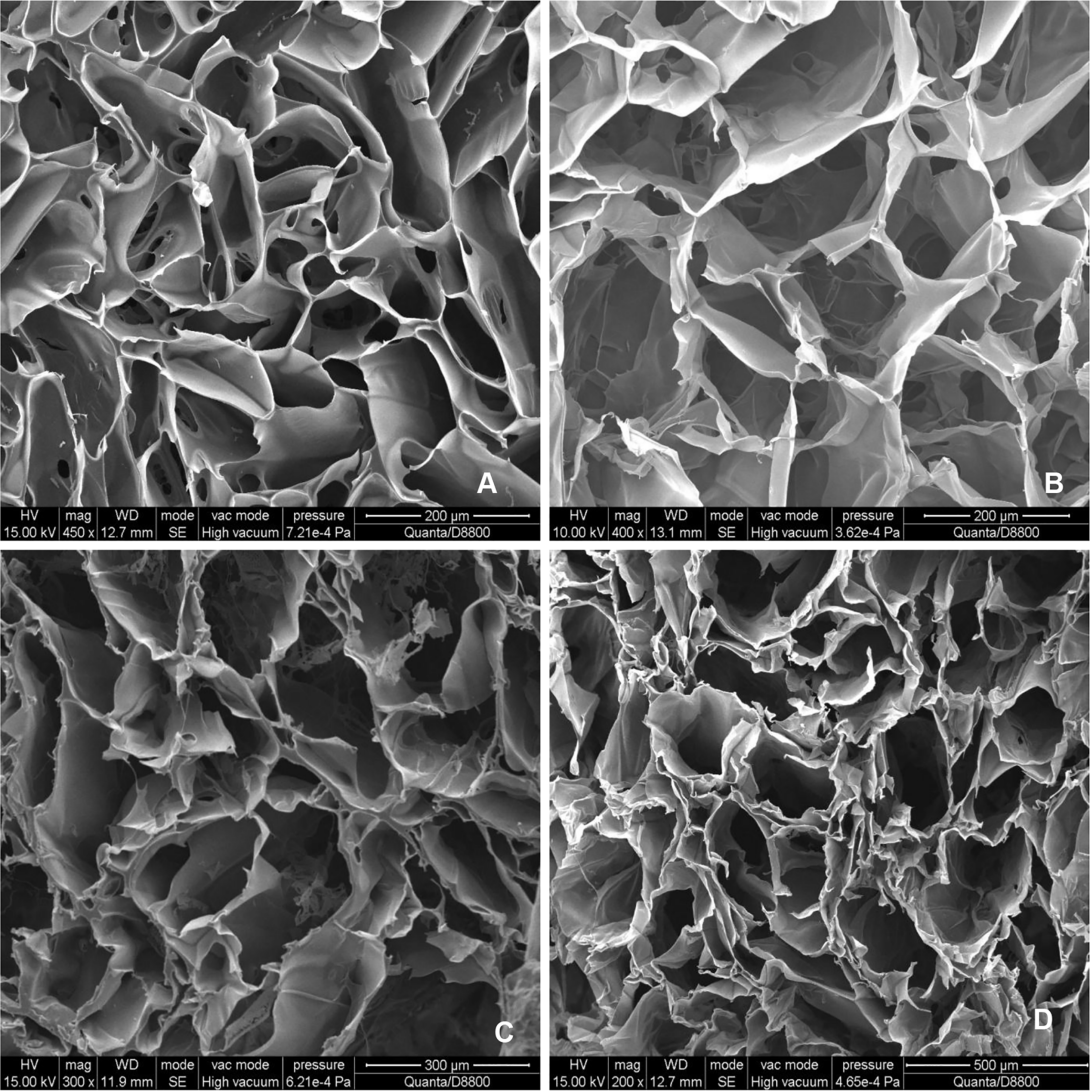
SEM images of SF/CS scaffold materials. (A) In the 20% SF-80% CS group, a porous network structure appeared. The thickness of the pore walls was uniform, yet the shape and size of the pores were not very regular, and the connectivity between the pores was poor. The mean pore diameter was 100±20.56 μm. (B) In the 40% SF -60% CS group, the pore size was uniform, and the pore walls were a single layer with a smooth surface and uniform thickness. The connectivity between the pores was good, and the pores themselves were round and relatively uniform with a mean pore size of 150±28.56 μm. (C) In the 60% SF-40% CS group, an irregular laminar curled structure appeared. The connectivity between the pores was relatively good. The mean pore size was 210±23.71 μm. (D) In the 80% SF-20% CS group, the pore size was large and the wall of each pore was not smooth, but composed of multiple irregular laminar structures. The curling was more notable than the 60% SF-40% CS group, and the pore shapes were not very uniform. The mean pore size was 300±23.43 μm.

Examination of the internal structure of composite materials with different SF/CS mass ratios under EM (Fig 3) revealed a porous network structure with pore sizes ranging from 100 to 350 μm and clear connection between pores. The scaffold morphology of the 40% SF-60% CS group was the best among all groups. It had a uniform pore size, single-layer pore walls, smooth surface, uniform thickness, and good connectivity between the pores. The pores are round with diameters of 150±28.56 μm.

#### (2) Porosity test result

The porosities were all greater than 90% (Table 1), except for pure SF which after lyophilization showed a rather loose structure and high dissolution loss rate. Thus, it was not possible to calculate the porosity for all other groups. High porosity helps the cell to adhere to the interior of the scaffold.

**Table 1:** Porosities of SF/CS composite scaffold with different mass ratios $\bar{x}$±*s* (*n* = 3).

SFratio:CSratio	Porosity(100%)
0%:100%	95.890±2.4
20%:80%	95.700±1.1
40%:60%	95.290±2.7
60%:40%	94.817±0.6
80%:20%	94.060±2.4
*F*value	2.538
*P*value	0.106

#### (3) Infrared analysis result

The pure SF amide I band was at 1654 cm^-1^, suggesting an α-helix structure. The amide II and amide III bands were at 1543 cm^-1^ and 1242 cm^-1^, respectively, indicating the presence of a random coil structure. Pure CS contains a characteristic sugar structure interconnected with β-(1,4) glycosidic bonds, and hence characteristic absorption peaks were observed at 1,154 cm^-1^ and 897 cm^-1^ [23].

**Fig 4:**
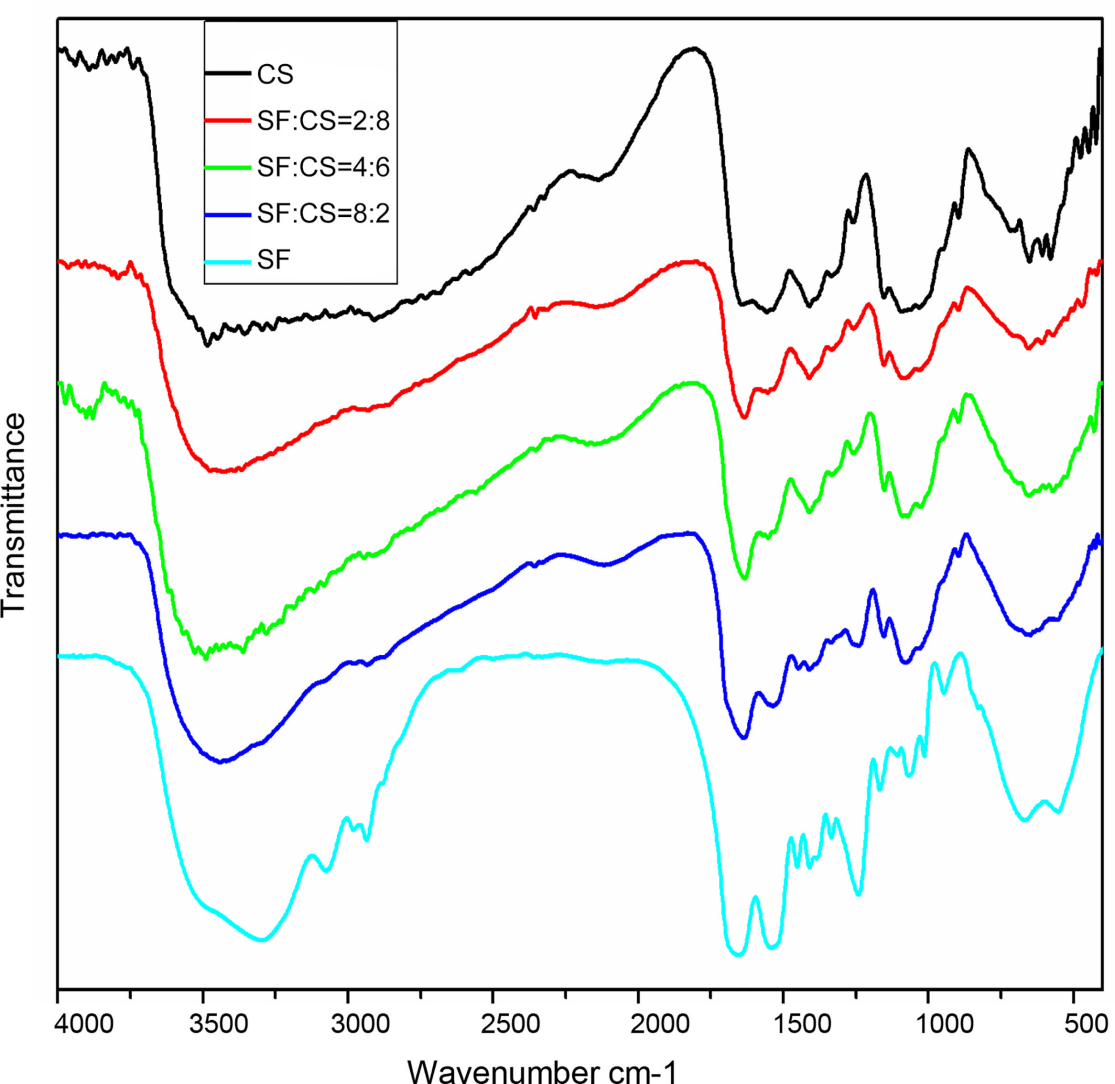
Fourier transform infrared spectra of SF/CS scaffold materials with different SF and CS mass ratios.

For the SF/CS scaffold materials, the absorption peak corresponding to the α-helix structure at 1654 cm^-1^ was reduced compared with pure SF. Of the four groups of composite SF/CS scaffold materials, the amide I bands were all at 1,637 cm^-1^-1,634 cm^-1^ representing β-sheets. This suggests that the addition of CS modifies the SF structure, making the unstable α helix/random coil structure change into a relatively stable β-sheet structure (Fig 4).

#### (4) XRD analysis result

**Fig 5:**
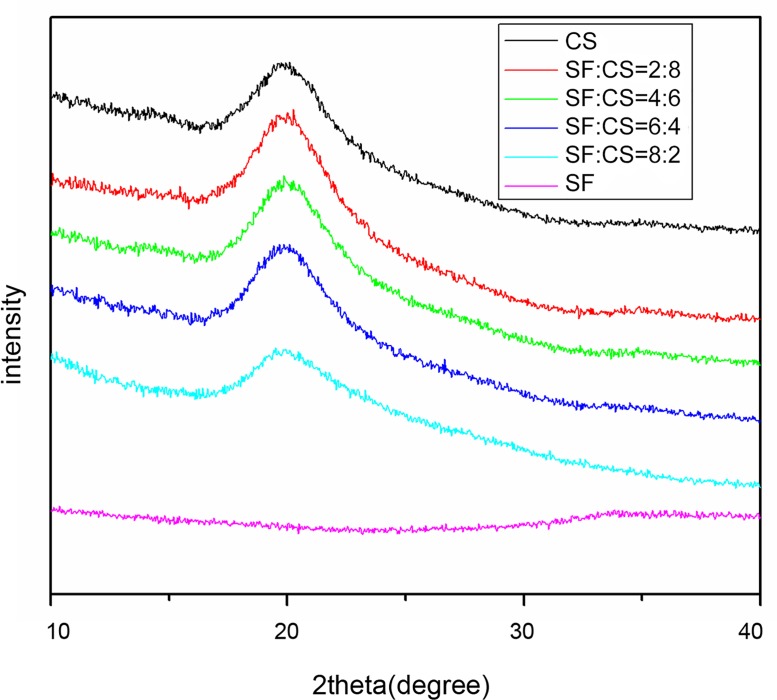
XRD spectra of SF/CS scaffold materials with different SF and CS mass ratios.

CS crystallinity was illustrated by the main bell-shaped peak with a 2*θ* of 20.10°. In comparison, the 2*θ* peak of SF was wide and dispersed suggesting low crystallinity of pure SF. This is consistent with the α helix/random coil structure of SF. In the SF/CS composite groups, the 2*θ* values were all 20.1° suggesting a crystalline form similar to that of CS. In other words, after adding CS, the crystallinity of the SF/CS composite increased compared with pure SF (Fig 5).

#### (5) EDS analysis results

**Fig 6:**
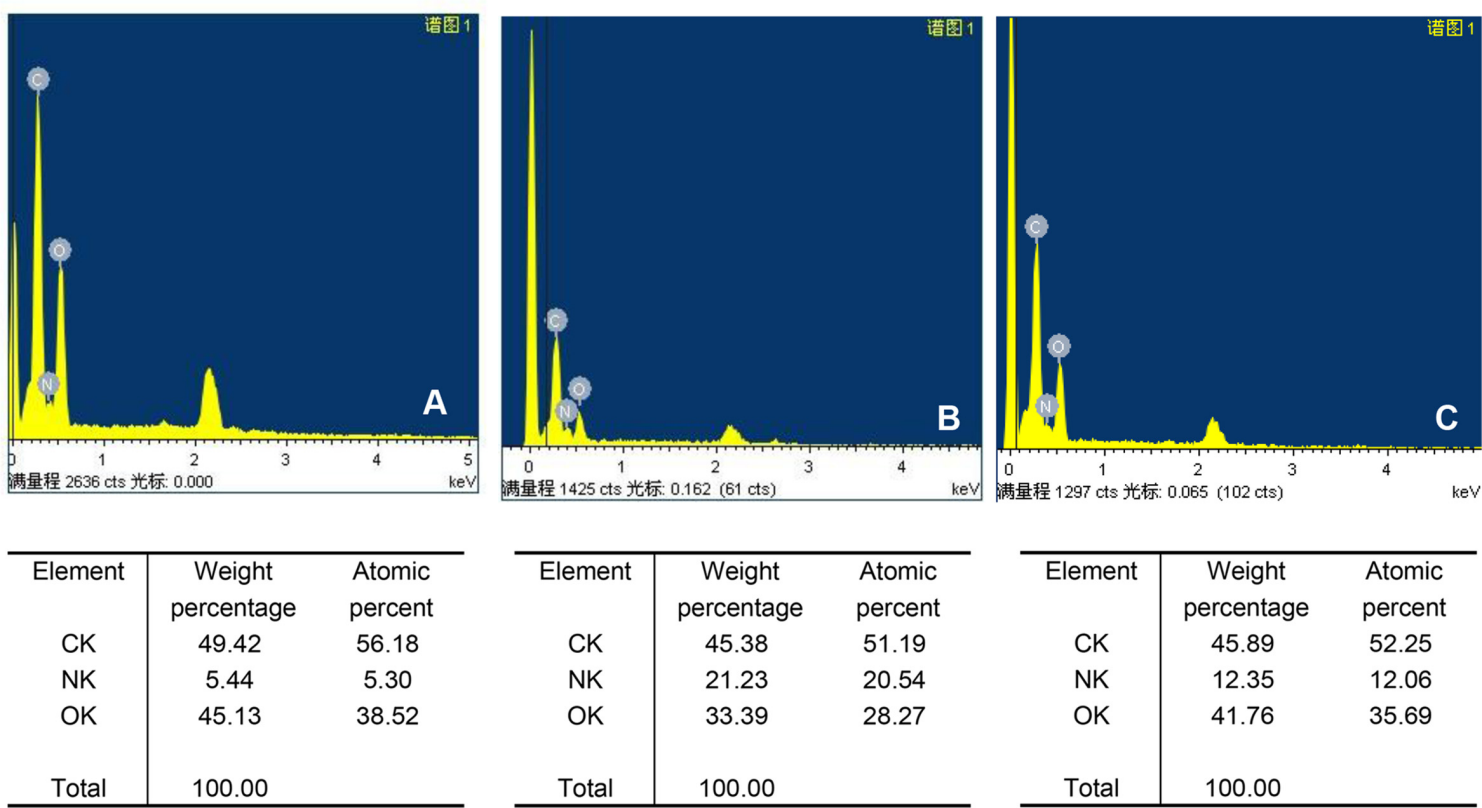
EDS spectrum of pure CS and pure SF and 40% SF-60% CS. The relative mass ratios of elements C, N, O of the 40% SF-60% CS group (C) were between that of pure SF (B) and pure CS (A). This suggests that only simple blending between SF and CS occurs and no new substance or element emerges.

By comparing Fig 6, we see that the relative mass ratios of C, N, O of the 40% SF-60% CS group (Fig 6C) are between that of pure SF (Fig 6B) and pure CS (Fig 6A). This suggests that SF and CS are simply blended with no new compounds. In Fig 6, peaks near 2 keV were found in all cases. This was because the EDS analysis was performed after adding surface gold and this peak represents gold.

### 2. Physical and chemical properties of the 3D porous SF/CS scaffold materials and their degradation

#### (1) The physical and chemical properties of the scaffolds

The physical and chemical properties of the scaffolds are illustrated in Table 2.

**Table 2:** Water absorption and swelling ratios of SF/CS composite scaffold with different mass ratios $\bar{x}$±*s* (*n* = 3).

Group	Water absorption (100%)	Swelling ratio (100%)
100% CS	1128.81±16.30	79.65±0.12^▲^
20% SF-80% CS	1069.83±30.56	70.44±1.13
40% SF-60% CS	1093.93±45.22	66.56±2.03
60% SF-40% CS	1112.82±28.12	57.40±2.15^▲^
80% SF-20% CS	1126.83±15.33	47.43±0.66^▲^
*F* value	2.034	1696.000
*P* value	0.165	0.000

Note:

^▲^vs. 40% SF-60% CS group and 20% SF-80% CS group.

*P*<0.05.

All scaffold groups absorbed water with a water absorption rate exceeding 1,000%. The swelling ratio of pure CS nearly reached 80%. As the CS content decreased, the swelling ratio also decreased. Thus, we conclude that the 20% SF-80% CS group and the 40% SF-60% CS group were better than the other groups due to their moderate swelling.

#### (2) Degradation test

Based on the results of the above experiment, the scaffold in the 40% SF-60% CS group was selected for this test. See Table 3.

**Table 3:** The pH values and degradation ratios of the 40% SF-60% CS group $\bar{x}$±*s* (*n* = 3).

Time (d)	pH value	Degradation ratio (100%)
0	7.563±0.0058	0.000
1	7.420±0.0000	6.597±0.1270
3	7.470±0.0100	9.787±0.1848
6	7.513±0.0058	10.910±0.1732
9	7.527±0.0058	12.373±0.2194
12	7.533±0.0058	13.637±0.2194
15	7.520±0.0100	14.430±0.2425
22	7.520±0.0100	15.793±0.2021
29	7.527±0.0058	16.540±0.2252
36	7.567±0.0058	17.020±0.0000
50	7.617±0.0058	17.913±0.2309
64	7.623±0.0058	18.247±0.2136

**Fig 7:**
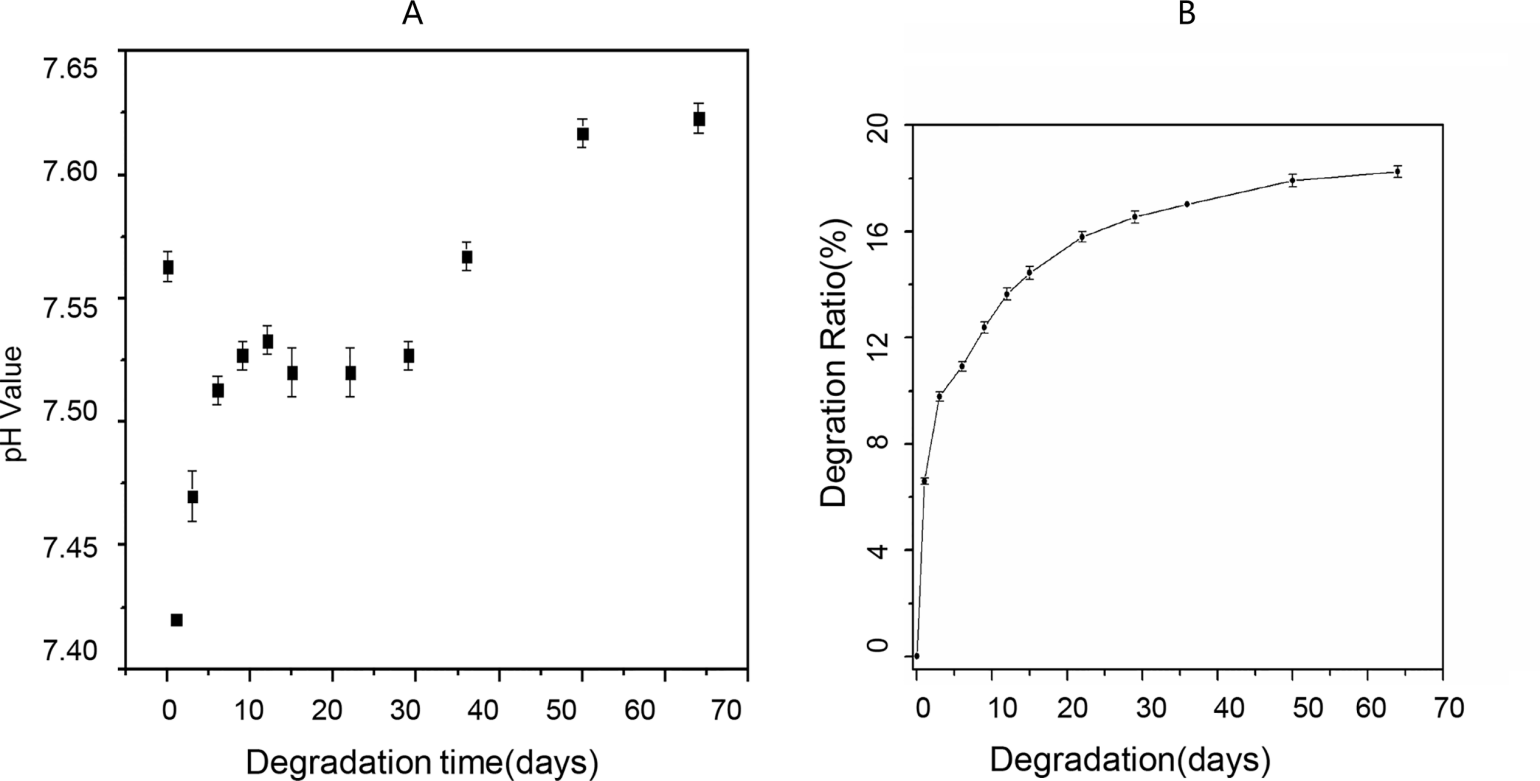
Change in the pH of the lysate and the degradation rate in the 40% SF-60% CS group. (A) pH fluctuated in a small range of 7.42~7.62; (B)The scaffold lost only 18.25% of its original weight after 64 days.

During the degradation process that lasted for two months, the pH fluctuated in a small range 7.42–7.62 (Fig 7A). In the first 12 days, the pH slowly increased, possibly due to the gradual degradation of the alkaline CS into the water. This continued until the pH reached 7.53. It then decreased perhaps because of degradation of acidic amino acids in SF. Afterwards, the pH value again slightly increased. The mass percentage of CS was higher than that of SF, and hence the degradation of the alkaline CS resulted in the slight pH increase.

The degradation rates of the scaffold material in the 40% SF-60% CS group over 64 days are shown in Fig 7B. In the first 15 d, the degradation rate was the fastest, and the scaffold material lost 14.43% of its original weight. In the next 21 d, the degradation rate slowed, and by day 36, a total of 17.02% of its original weight had been lost since the start of the experiment. In the last 28 d, the degradation rate became even slower—by 64 d the scaffold had been lost 18.25% of its original weight since the start of the experiment.

### 3. The biocompatibility and osteogenic properties of the 3D SF/CS porous scaffold

#### (1) The biocompatibility between MG-63 cells and the scaffold material

**Fig 8:**
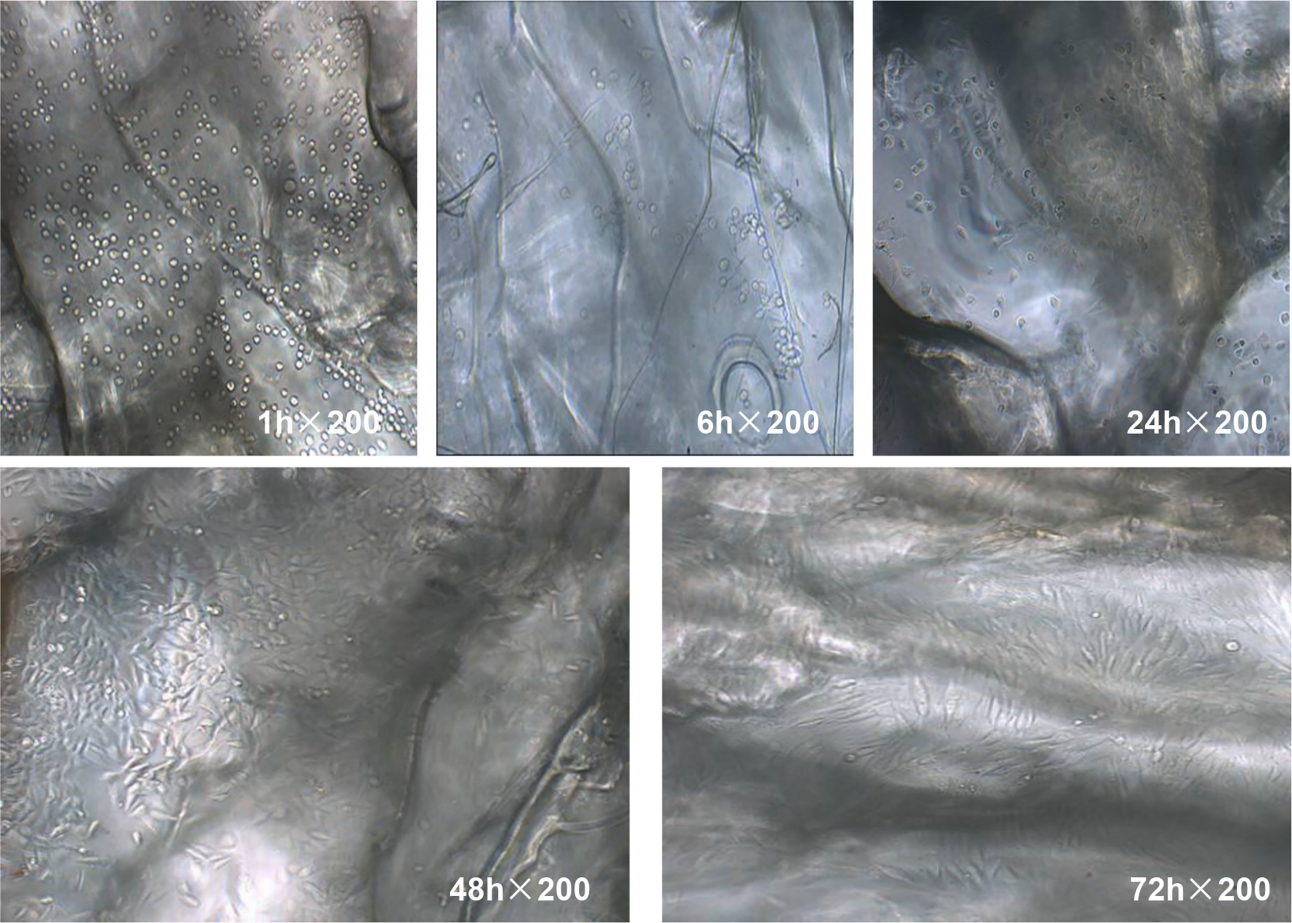
MG-63 cell growth on the scaffold. At 6 h after inoculation, the cells started to adhere to the walls of the scaffold material; at 24 h the adhesion completed and the cell volumes increased. At day 2, the cells became spindle-shaped or triangular, showing junctions between the growth projections; at 3 d the cells were tightly connected to each other, and the walls and pores of the scaffold material were all covered with cells.

**Fig 9:**
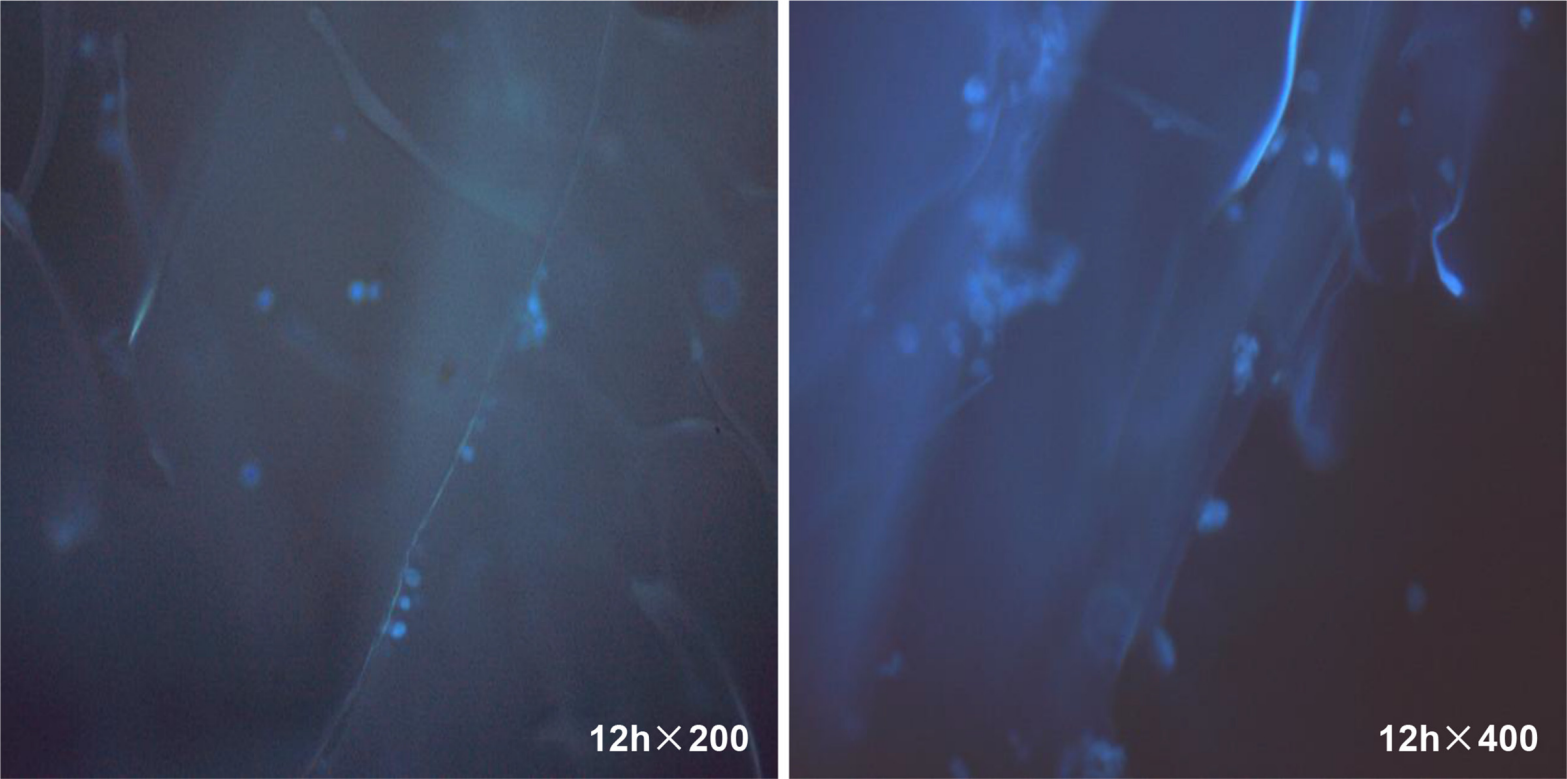
Fluorescence image showing MG-63 cell adhesion to the scaffold material. At 12 h after inoculation, the cells uniformly adhered to the pore walls of the scaffold.

Freshly seeded cells were transparent, spherical, and uniformly distributed on the scaffold. At 6 h after inoculation, they started to adhere to the walls of the scaffold material (Fig 8). At 12 h, a large number of cells adhered to the scaffold (Fig 9). After 24 h, the majority of cells had completed adhesion and cell volumes increased; a small number of cells showed growth projections. Two days after inoculation, the cells became spindle-shaped or triangular with notable projections, and junctions between the growth projections began to form. At 3 d after inoculation, the cells were tightly connected to each other, and the walls and pores of the scaffold material were completely covered with cells.

#### (2) Cell adhesion rate

**Fig 10:**
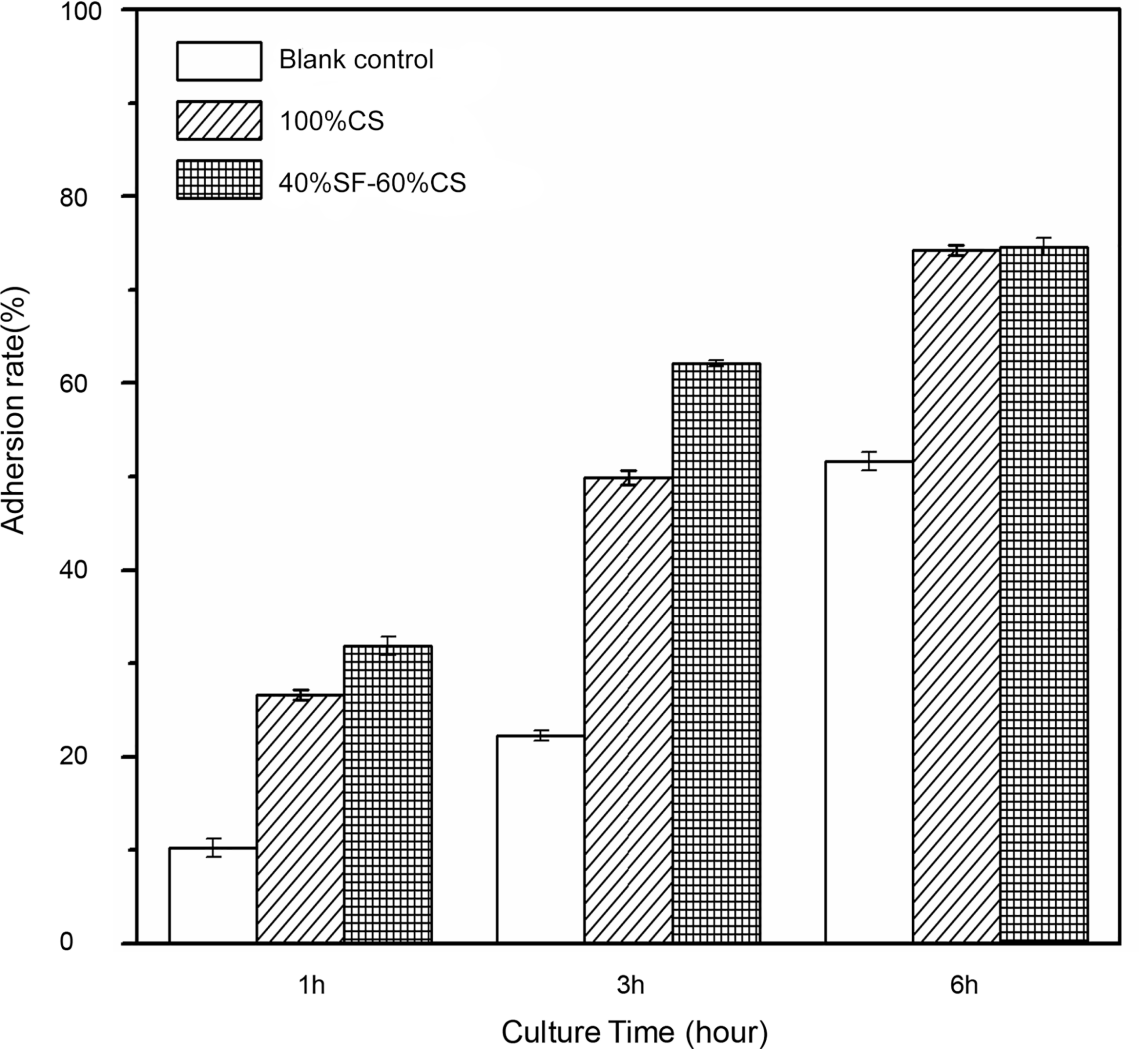
Adhesion rates of MG-63 cells at different time points. The adhesion rates of both experimental groups were significantly higher than that of the blank control side group. At 1 h and 3 h, the adhesion rate of the 40% SF-60% CS group was significantly higher than that of the 100% CS group; at 5 h, the two groups did not differ significantly.

There were more adhered cells in both the 100% CS group and the 40% SF-60% CS group than the blank control group. At 1 h, the adhesion rate of the MG-63 cells on the scaffold material in the 40% SF-60% CS group exceeded 30%, and at 3 h, it was greater than 60%. This was significantly higher than the 100% CS group. At 6 h, the adhesion rates of both experimental groups were close to 75% (Table 4, Fig 10).

**Table 4:** Adhesion rates of MG-63 cells at different time points ($\bar{x}$±*s*, *n* = 3).

Group	Time			*F* value	*P* value
	1 h	3 h	6 h		
Control group	10.250±0.94	22.250±0.57	51.625±0.99	1851.000	<0.001
100% CS	26.625±0.57^▲^	49.875±0.78^▲^	74.258±0.56^▲^	4074.000	<0.001
40% SF-60% CS	31.875±0.94^▲^ ^★^	62.125±0.36^▲^ ^★^	74.627±0.95^▲^	2257.000	<0.001
*F* value	542.822	3483.000	711.445	142.707^#^	
*P* value	<0.001	<0.001	<0.001		<0.001^#^

Note:

^▲^ significantly different from the scaffold in the control group (*P*<0.001).

^#^ interaction effect.

^★^ significantly different from the 100% CS group (*P*<0.001).

#### (3) Hoechst staining result

**Fig 11:**
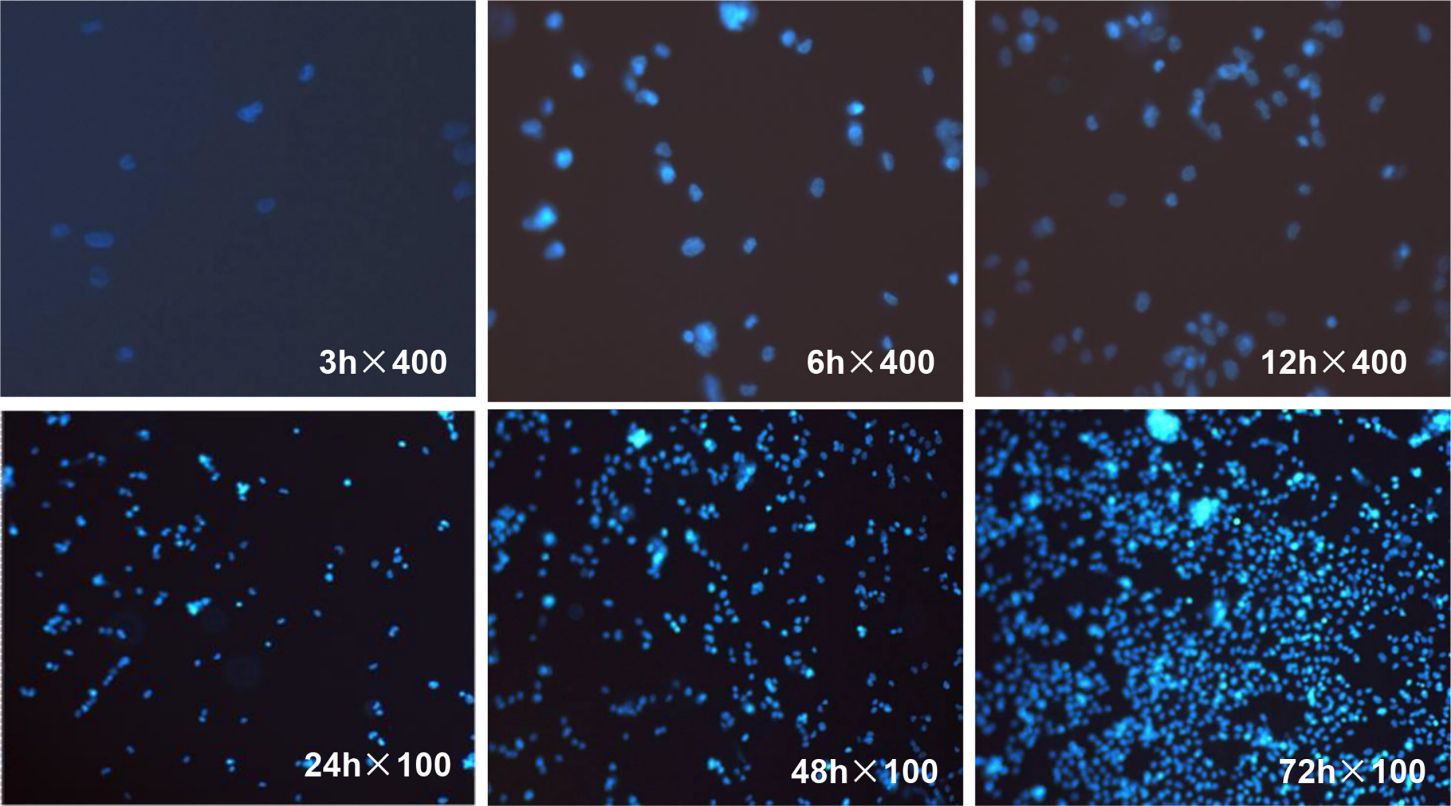
Fluorescence image showing MG-63 cell growth on the scaffold material. At 3 h a very small number of cells adhered to the scaffold, but at 6 h and 12 h the number of adhered MG-63 cells increased substantially. At 1 d the cell nuclei began to show dispersed and uniform blue fluorescence. At 2 d the fluorescence intensity increased substantially, and at 3 d a granular fluorescence was observed due to increased cell density.

Hoechst staining revealed the adhesion and proliferation of cells on the scaffold materials. At 3 h only a very small number of cells adhered to the scaffold, but at 6 and 12 h the adhesion rates of MG-63 cells increased. At 100X magnification we noted that after 1 d the cell nuclei began to show dispersed and relatively uniform blue fluorescence, and at 2 d the fluorescence intensity increased substantially. On day 3, granular fluorescence was observed due to increased cell density (Fig 11).

#### (4) Cell proliferation

**Fig 12:**
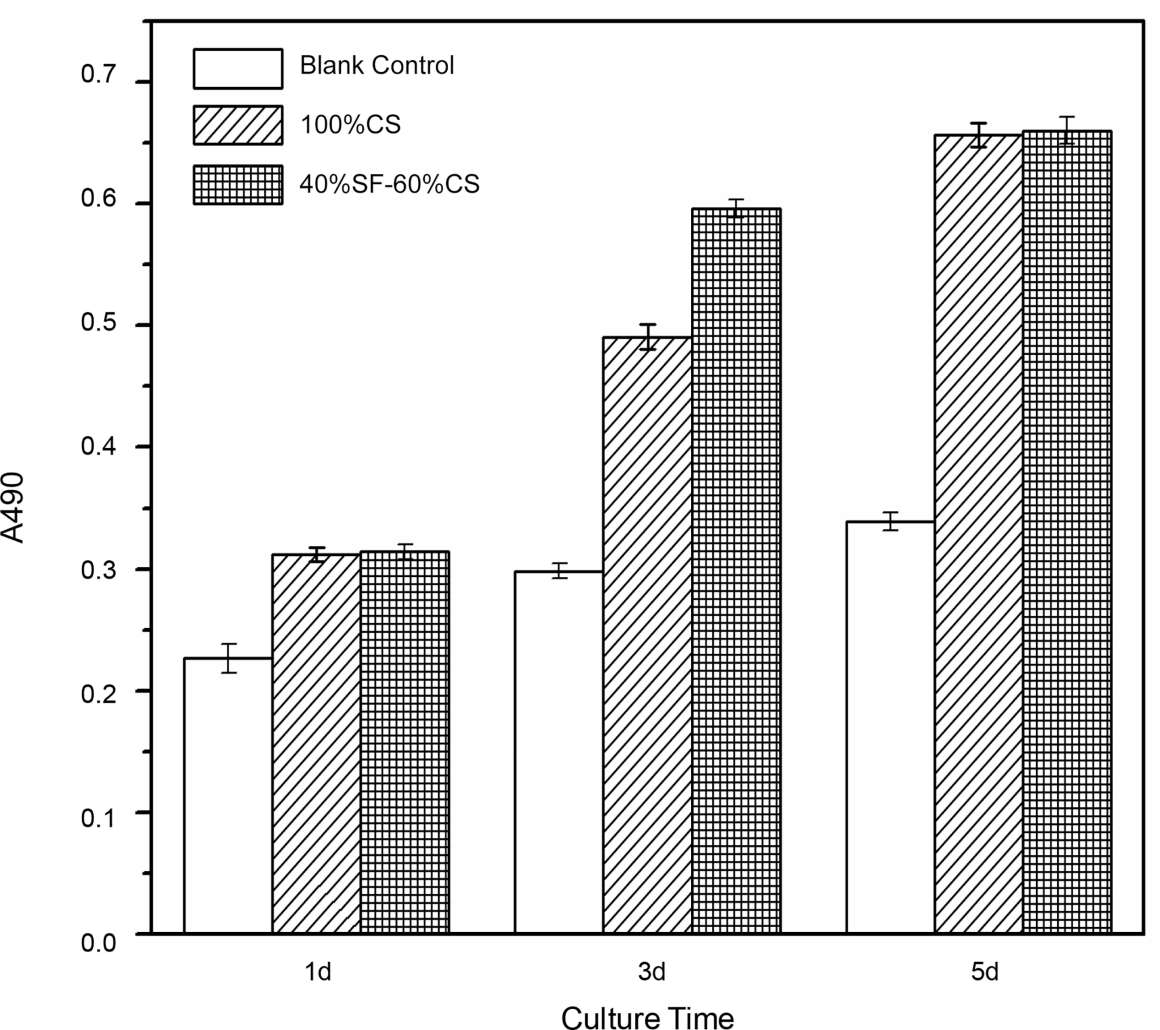
MG-63 cell proliferation revealed by OD values in different scaffold groups and at different time points. The MG-63 cell proliferation rates of both experimental groups were significantly higher than that of the blank control slide group. On day 3, the proliferation rate of the 40% SF-60% CS group was significantly higher than that of the 100% CS group. On day 1 and 5, the two experimental groups did not differ significantly.

The MG-63 cell proliferation rates in the 100% CS group and 40% SF-60% CS group were significantly higher than that in the blank control slide group. On day 1, the OD values of both groups were about 0.31, and the two did not differ significantly. On day 3, the proliferation rate in the 40% SF-60% CS group was significantly higher than that in the 100% CS group. On day 5, the cells still proliferated, but the speed was slower. The proliferation rate no longer differed significantly between the two experimental groups (Table 5, Fig 12).

**Table 5:** MG-63 proliferation rates at different time points ($\bar{x}$±*s*, *n* = 6).

Group	Time			*F* value	*P* value
	1 d	3 d	5 d		
Control group	0.226±0.012	0.298±0.006	0.339±0.008	252.447	<0.001
100% CS	0.312±0.006^▲^	0.491±0.010^▲^	0.657±0.010^▲^	225.000	<0.001
40% SF-60% CS	0.314±0.006^▲^	0.596±0.008^▲^ ^★^	0.660±0.011^▲^	4.851	<0.05
*F* value	216.317	2036.000	2262.000		
*P* value	<0.001	<0.001	<0.001	436.443^#^	<0.001^#^

Note:

^▲^ significantly different from the scaffold in the control group (*P*<0.001).

^#^ interaction effect.

^★^ significantly different from the 100% CS group (*P*<0.001).

#### (5) ALP activity

**Fig 13:**
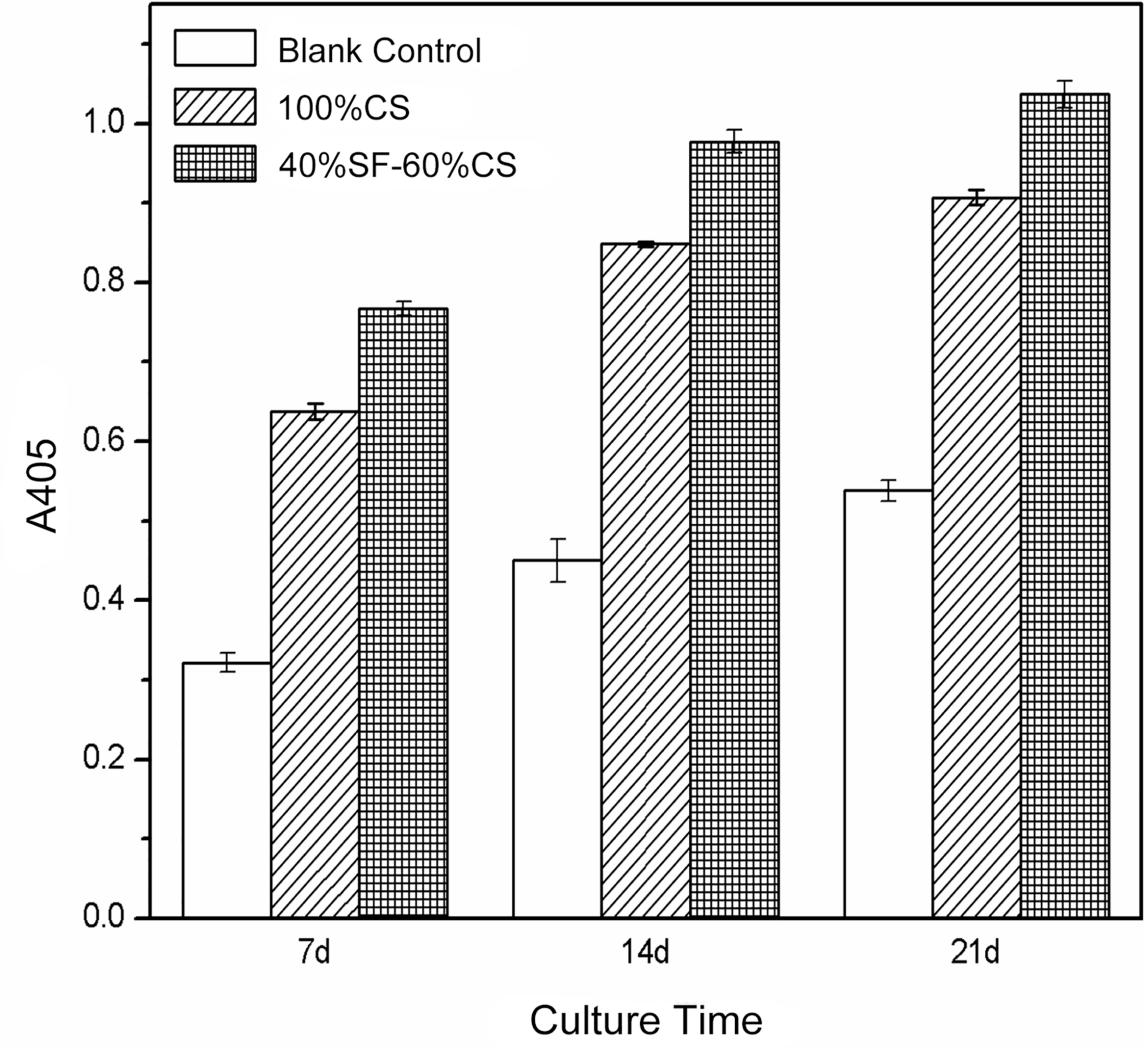
Secretion of ALP by MG-63 cells in different scaffold groups and at different time points. ALP secretion by MG-63 cells in the two experimental groups was significantly stronger than that in the blank control group. On day 7, 14, and 21 the 40% SF-60% CS group facilitated osteogenesis significantly more than the 100% CS group.

The 100% CS group and the 40% SF-60% CS group both promoted ALP secretion by MG-6 cells. The ALP activities in these two groups were both significantly higher than that in the blank control group (*P*<0.001). At 7 d, 14 d, and 21 d the two experimental groups also differed significantly in ALP activity (Table 6, Fig 13).

**Table 6:** Activities of ALP secreted by MG-63 cells on different scaffolds at different time points ($\bar{x}$±*s*, *n* = 6).

Group	Time			*F* value	*P* value
	7 d	14 d	21 d		
Control group	0.322±0.011	0.450±0.027	0.538±0.013	202.915	<0.001
100% CS	0.638±0.010^▲^	0.849±0.003^▲^	0.907±0.009^▲^	1823.000	<0.001
40% SF-60% CS	0.767±0.009^▲^ ^★^	0.978±0.008^▲^ ^★^	1.037±0.017^▲^ ^★^	636.731	<0.001
*F* value	2980.000	813.988	2208.000	17.198^#^	<0.001^#^
*P* value	<0.001	<0.001	<0.001		

Note:

^▲^ significantly different from the scaffold in the control group (*P*<0.001).

^#^ interaction effect.

^★^ significantly different from the 100% CS group (*P*<0.001).

#### (6) The ability to form calcified nodules

**Fig 14:**
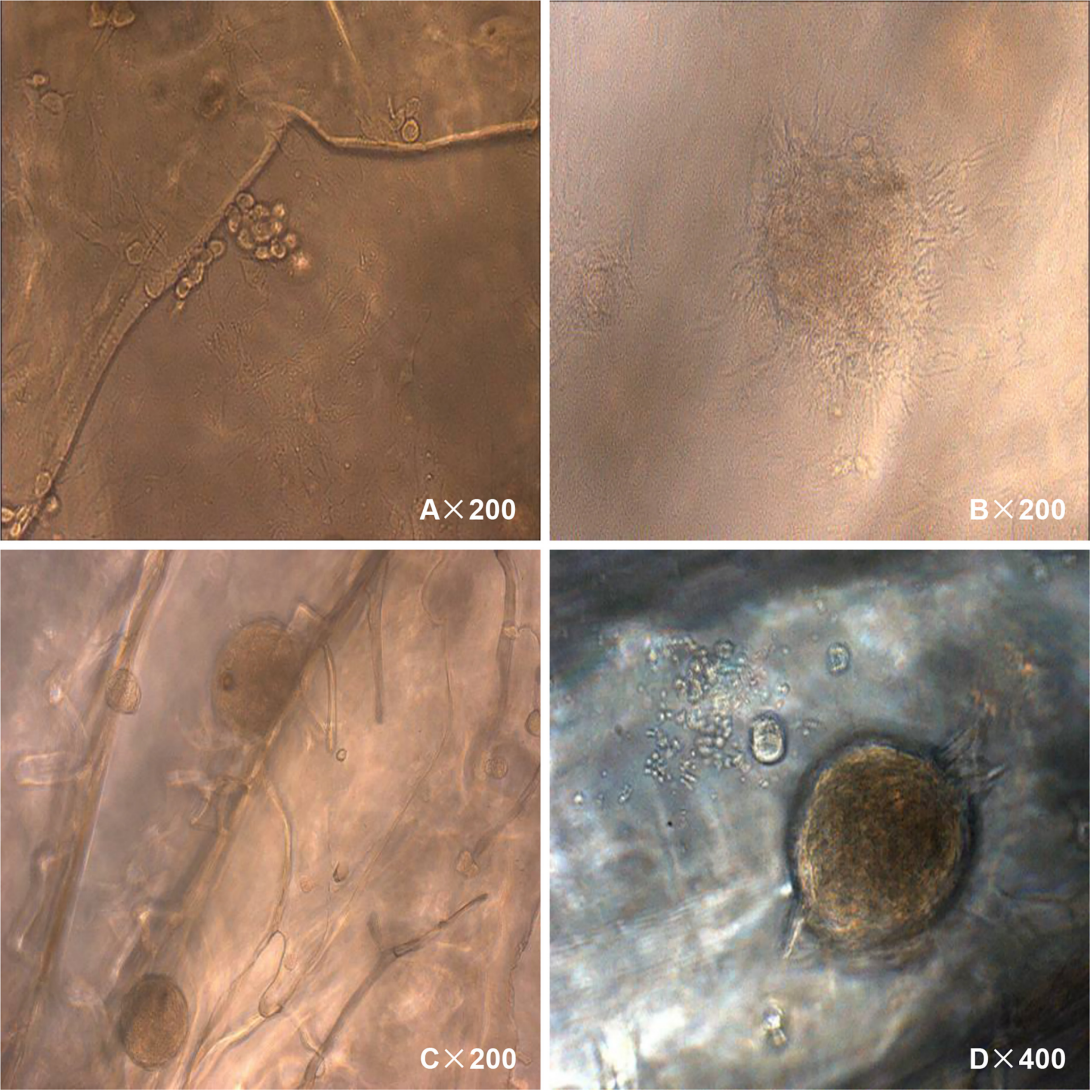
The mineralization of MG-63 cells on the scaffold. (A) Osteoblasts secreted a spherical matrix, which clustered together. (B) Osteoblasts grew in multi-layers and clustered into groups. (C and D) The matrix secreted by the osteoblasts was mineralized, forming calcified nodules.

On day 7, light microscopy revealed that the 100% CS group and the 40% SF-60% CS group had lumpy, brown-black calcified nodules in the pore wall of the scaffold. The nodules increased in number as the incubation time increased. At 12 d, nodules started to appear in the 40% SF-60% CS group, but not until day 16 in the 100% CS group. After the cells grew and gradually formed a single layer, their proliferation rate decreased. The cell body increased, followed by secretion of bone extracellular matrix. By light microscopy we observed that the osteoblasts had a spherical matrix, and these spherical matrices tended to form clusters (Fig 14A).

From 7–10 d, osteoblasts with multi-layer growth grouped together (Fig 14B). The cells grew in colonies, and the cell boundaries of the cells in the center of these colonies were not clearly visible. For cells growing at the periphery, intertwined protrusions were observed. These colonies on the scaffold materials appeared three dimensional, and the focal length of the microscope had to be adjusted to clearly visualize different planes. Meanwhile, the matrix secreted by the osteoblasts began to be mineralized over time, forming calcified nodules (Fig 14C and 14D). Over the entire mineralization process, all steps—including cell clustering, matrix secretion and the final mineralization—occurred later in the 100% CS group than the 40% SF-60% CS group.

## Discussion

In tissue engineering, there are many methods to prepare scaffolds including particulate leaching, microsphere sintering, gas foaming and freeze-drying. Particulate leaching proposed by Mikos et al. [24,25] prepares porous scaffolds by separating particles with filtration. For example, substances that are water-soluble but insoluble in organic solvent, such as sodium chloride and sugar, can be used as pore-forming agents in the particulate leaching method. Based on the different solubility and volatility of different substances, the pore-forming agent can be removed to form a porous scaffold. The drawback of this method is that the connection between the pores is relatively poor, and it is difficult to completely remove the residual pore-forming agent.

Sintering microspheres at high temperature followed by cooling also forms porous scaffolds. Its drawback is that organic solvent must be used during the preparation and is difficult to remove. Scaffolds made by sintering also have low porosity. Gas foaming, also known as pressure quench foaming [26], uses carbon dioxide as the pore-forming agent. The advantage of this technique is that no organic solvent is used and hence residual organic solvent is no longer a problem. However, the formed pores often have a closed structure, and the pore size is relatively small.

Lyophilization (freeze-drying) [27,28] is the most commonly used method to make scaffolds. After quickly freezing the material, high vacuum sublimates the ice, eventually drying the material. The advantage is that no organic matter is involved, and the use of a pure physical method maximally protects the properties of the raw materials while forming a porous, sponge-like structure.

We used freeze-drying in this study. First, the SF aqueous solution and CS solution of fixed concentrations were blended at various mass ratios. After the first lyophilization, followed by treatment with anhydrous methanol/10% sodium hydroxide and then with the EDC/NHS crosslinking agent, the material was washed and subjected to another lyophilization to prepare the SF/CS composite scaffold. Zhang’s group [29] reported that crosslinking SF/hydroxyl CS using alcohol was not as effective as the drug Genipin. Qazvini and colleagues [30] reported that EDC/NHS was an efficient non-toxic crosslinking agent. Here we show that after the initial treatment with methanol/10% sodium hydroxide, the dissolution loss rate was still relatively high, possibly because this treatment only neutralized the acetic acid in CS and denatured the proteins. Hence, crosslinking via EDC/NHS was applied to give a stable scaffold. Indeed, stability in aqueous solution and hardness were both enhanced.

The scaffold material used for tissue engineering should have an appropriate pore size for cells to attach and migrate. In the present study, mixing SF and CS offered a network structure with pore sizes ranging from 100 μm to 350 μm with interconnected pores. It has been reported that uniform and regular pores with high porosity can improve the mechanical properties of the scaffold [31]. As the CS content decreased, the pore size increased. EM showed that a few pores with diameters above 400 μm could be observed. Furthermore, when the CS content was high, the connectivity rate was lower than when the CS content was low. When SF content was high, the internal structure of the scaffold material had laminar pore walls. Thus, by varying the mass ratio of the two different components, the morphology and size of the pores can be controlled.

In a recent study [32], SF-collagen scaffolds were compared to SF-CS scaffolds and data indicated that SF-collagen was preferable to SF-CS for cell growth. However, the porosity of the SF-CS scaffold was lower than the SF-collagen scaffold, and pore sizes were smaller for the SF-CS scaffolds (average 76 μm). Cell growth is highly dependent on pore size, and the size of the pores in the SF-CS scaffold were smaller than the pores we observed in our 40% SF-60% CS scaffold. This underscores the importance of controlling parameters involved in the processing of the scaffold.

Each protein has a unique conformation. The animal protein SF is no exception. Many studies have shown that its mechanical properties depend on its conformation. Research on protein conformation typically focuses on the infrared positions of the amide I band, amide II band and the amide V band. As early as 1950, Elliott and coworkers [33] proposed that the absorption peak of thalidomide I band at 1,660–1,650 cm^-1^ belonged to the α-helix structure, and the peak from 1,640 cm^-1^-1,630 cm^-1^ was the β-sheet conformation. In addition, the characteristic peaks of the amide II band and the amide III band in general are located near 1540 cm^-1^ and 1240 cm^-1^, respectively. These correspond to the random coil structure of SF.

We found that the conformation of pure SF was dominated by α-helix/random coil structures. This might be the reason why pure SF was a powder after lyophilization, difficult to grip, and with poor mechanical properties. For composite SF/CS scaffold materials, the four groups with different mass ratios all showed an amide I band at 1637 cm^-1^-1634 cm^-1^, indicating β-sheets. This suggests that the addition of CS modifies the structure of SF and makes the α-helix/random coil structure unstable, effectively transforming into the relatively stable β-sheet structure. This was in agreement with the XRD data. After adding CS, the crystallinity of the scaffold in the various SF/CS groups increased compared with pure SF, and the intensity of the crystallization peak of these blended materials was not lower than that of pure CS, perhaps because soaking in crosslinking agent intensified the crystallization. No chemical reaction or generation of new substances was observed after soaking the scaffold materials in the crosslinking agent EDC/NHS, suggesting that crosslinking SF/CS scaffold materials using EDC/NHS can offer a stable product in aqueous solutions without affecting the blending between the two components.

Scaffold materials with superior properties must have good water absorption. If the scaffold material absorbs sufficient cell culture medium, the cells adhering to the walls can obtain ample nutrition and proliferate. However, the swelling ratio also needs to be considered. If the water absorption rate is too high, the resulting swelling may deform the scaffold material and shrink the original pores or even cause the pores to disappear. This is detrimental to cell growth.

CS has excellent hydrophilicity, and contains large amounts of reactive amino and carboxy groups. After treatment with methanol and a crosslinking agent, the internal structure of the SF often exhibits a silk II structure. This causes the internal peptide chains to be arranged in an orderly manner and increases the ability of SF to resist external forces. Thus, only mild swelling occurs without dissolution of SF. When SF is mixed with CS, the active amino groups in CS form hydrogen bonds not only with the carboxyl groups in CS, but also with the carboxyl groups in SF. This causes the water-soluble α helix/random coil structure of pure SF transform to a stable β-sheet structure. This feature and stabilization by secondary treatments with anhydrous methanol/10% sodium hydroxide and EDC/NHS crosslinking agent limit the degree of swelling. The 20% SF-80% CS group and the 40% SF-60% CS group had moderate swelling ratios. In summary, we selected the 40% SF-60% CS group as the ideal sample for the degradation test.

Scaffold materials should also have an appropriate degradation rate [34]. During *in vitro* cell growth, scaffold materials are being constantly degraded, and the degradation rate should match the growth rate of the cells [35, 36]. In other words, the degradation rate of the scaffold should match the rate at which new tissues are generated. When the new tissues are formed, the scaffold material should completely degrade and safely be absorbed by the body.

Horan’s group [37] placed SF in PBS to observe the degradation reaction, and found that in ten days the SF mass did not change. In the present study, the initial rate of degradation of the scaffold material was the fastest perhaps because of CS degradation. This was also consistent with the changes in the pH of the SBF containing the scaffold materials. In our *in vitro* degradation test that lasted 64 d, the pH of the degradation fluid fluctuated within a narrow range of 7.42–7.62 corresponding to the pH value of *in vivo* microenvironment. The degradation rate was very stable, which suggests that the scaffold material in the 40% SF-60% CS group may be a good candidate for future *in vivo* experiments.

We found that MG-63 cells grew well on the scaffold, yet the adhesion rate was relatively low. This is probably because the scaffold did not occupy the entire well area, and cells attached to the available surface surrounding the scaffold, causing the measured value of adhesion to be relatively small.

The Hoechst stain is a nuclear dye with membrane permeability and the ability to bind to live or fixed cells, so it safe and reliable for labeling live cells. ALP expression is an early sign of osteoblast maturation and indicates *in vitro* osteoblast differentiation into mature bone cells and matrix mineralization [38]. Calcified nodules are formed after a certain period of osteoblast proliferation and represent the maturation of osteoblast growth and differentiation. So, they are an important marker for identification of osteoblasts. Formation of calcified nodules directly reflects the ability of osteoblasts to induce matrix mineralization.

Osteoblasts subjected to continuous *in vitro* culture can form calcified nodules. Bellow’s group [39] showed that calcified nodules were formed by osteoblasts after 2–3 weeks of proliferation and differentiation. Calcified nodules are a major sign of osteoblast maturation. To form calcified nodules, the multi-layer growth of osteoblasts must go through three stages: quick proliferation, maturation of extracellular matrix, and matrix mineralization. Mineralization is only possible after entering the final maturation stage [40, 41].

Here, MG-63 cells grew well on 40% SF-60% CS scaffold. The adhesion rate and proliferation rate were both significantly enhanced compared with the blank control slide group. The 40% SF-60% CS group also promoted secretion of ALP by MG-63 cells significantly more than the blank control group and the 100% CS group. Furthermore, the 40% SF-60% CS group promoted cell clustering, matrix secretion and final mineralization significantly earlier than the 100% CS group.

## Conclusion

SF and CS are both natural polymer materials with good biological properties. After the two are blended, they mutually modify each other. Here we prepared scaffolds with freeze-drying and immersion cross-linking. Infrared analysis and XRD analysis confirmed the changes in the micro-properties of the composite material compared with each component. Further tests on the physical and chemical properties showed that the 40% SF-60% CS group was the best combination. *In vitro* degradation experiments lasting 64 d were performed on 40% SF-60% CS scaffold material, and the degradation rate was found to be stable with a pH neutral lysate corresponding to the pH of *in vivo* microenvironment. *In vitro* experiments suggested that after modification, the 40% SF-60% CS scaffold material has good biocompatibility, and can promote the biological function of osteoblasts. Hence, it is a good scaffold material for bone tissue engineering.
